# Climbers of the Estação Ecológica de Assis, State of São Paulo, Brazil: floristics and identification keys

**DOI:** 10.3897/phytokeys.99.13659

**Published:** 2018-05-21

**Authors:** Nicácio Ribeiro Neto, Raquel Aparecida Ronqui, Letícia Chedid Seidinger, Renata Giassi Udulutsch

**Affiliations:** 1 Universidade Estadual Paulista Júlio de Mesquita Filho (UNESP), Faculdade de Ciências e Letras de Assis, 19806-900, Assis, São Paulo, Brazil; 2 Universidade Estadual Paulista Júlio de Mesquita Filho (UNESP), Instituto de Biociências, 13506-900, Assis, São Paulo, Brazil

**Keywords:** cerradão, biodiversity, forested savannah, liana, seasonal forest, vine

## Abstract

Climbers are an important life form in the understory and canopy of tropical forests. They are characterised by constant root connection to the ground and use of other species, mainly trees, as support in their search for higher light. In addition, they have an important role in ecological succession in forest clearings, as they are able to develop rapidly in these environments. Climbers can have high species richness in the biomes in which they are present. Since climbers are of little economic importance, they are usually cut down without regard for their contribution to maintenance of biodiversity and to the structure of the forest. Floristic studies of climbers in Brazil are still scarce and more research is needed. The goal of our research was to develop a floristic survey and identification keys for the climbers of the Estação Ecológica de Assis (EEA) in the cerrado biome of São Paulo state, Brazil. Sampling was carried out every two weeks over ten months, along trails and edges of forest within the EEA. Identification keys were built based on vegetative characters. Thirty-two climber species, belonging to 24 genera and 13 families were recorded. The families with the largest number of species were Bignoniaceae (8 species), Malpighiaceae (5), Apocynaceae (3) and Smilacaceae (3). The richest genera were *Fridericia* (4 species), *Banisteriopsis* (3) and *Smilax* (3). The number of species recorded for the forest fragment reveals the important role of climbers in the diversity of forested savannahs (“cerradões”) in the State of São Paulo.

## Introduction

Climbers are an important life form in the understory and canopy of tropical forests. They are characterised by having constant root contact with the ground (Hegarty 1991, Udulutsch et al. 2004) and, by using other plant species, primarily trees, as supporting structures, they search for more light (Gentry 1991), regardless of whether they are woody lianas or herbaceous vines (Udulutsch et al. 2010). The climbing habit has appeared independently several times during the evolution of phanerogams (Burnham 2015) and may be responsible for originating modified organs specialised for climbing (Isnard and Silk 2009, Angyalossy et al. 2015).

With a reduced mechanical demand, climbers use other means of support and wood lianas can thus exhibit peculiar stem anatomy, related to the climbing habit, such as variation in cambium activity, leading to different anatomical patterns (Isnard and Feild 2015), abundant parenchyma and fewer fibres than seen in self-supporting woody plants (Brandes and Barros 2008).

Lianas comprise about 25% of the woody species of tropical forests (Schnitzer and Bongers 2002). Moreover, in some forests, especially those on the banks of rivers in the Amazon drainage, lianas may contribute up to 44% of the woody species and have densities as high as 51 species per hectare (Pérez-Salicrup et al. 2001). Lianas also have an important role in succession in clearings because they develop quickly under high light (Sanches and Valio 2006, Vieira and Scariot 2008).

Traditionally, climbers are seen as pests, not only because they do not produce wood useful for the timber industry, but also because they interfere with the production of timber, therefore their importance for local sustainability is disregarded (Engel et al. 1998, Lopes et al. 2008). Since climbers contribute significantly to maintaining the diversity and structure of a forest (Gentry 1991), there is a need for studies that focus on characterisation, conservation and management of lianescent species.

Some authors (Putz 1984, Morellato and Leitão Filho 1995, Tabanez and Viana 2000) have emphasised the importance of studying climbers, with the goal of improving management and conservation of forest fragments. A problem in the management and maintenance of forest fragments can be the proliferation or invasion by weedy climbers and, since climbing plants can interfere with arboreal regeneration, their presence is considered as detrimental to trees (Putz 1984). Several studies have demonstrated that lianas have a negative effect on tree species, suppressing the growth in diameter and increasing the risk of death due to excessive weight on the trees that support them (Schnitzer et al. 2000, Phillips et al. 2002, 2005, Schnitzer and Bongers 2002, Malizia and Grau 2006, Van der Heijden et al. 2008, Ingwell et al. 2010, Visser et al. 2017). Climbers also contribute significantly to the leaf biomass of tropical forests, however, because of the high ratio of leaf to stem (Gentry 1983), the phenology of climbers can be complementary to that of other plant life forms, resulting in continuous nectar, pollen and fruit production for wildlife (Morellato and Leitão Filho 1996, Tibiriçá et al. 2006).

Long-term monitoring studies in Central America and the Amazon (Oliveira et al. 2008) demonstrate that liana abundance is changing, with this life form becoming more dominant. In a review study on liana abundance and biomass, Schnitzer and Bongers (2011) showed that several studies supported the pattern of increasing liana abundance and biomass in American tropical (e.g. Phillips et al. 2002, Wright et al. 2004, Chave et al. 2008) and subtropical forests (Allen et al. 2007), whereas other studies from Africa do not (e.g. Caballé and Martin 2001). However, more monitoring plots throughout the tropics are needed to confirm the mechanisms involved in this process (Schnitzer et al. 2015).

Despite their acknowledged importance from both floristic and ecological perspectives, climber species are neglected and are one of the least studied life forms of forest ecosystems. One reason for this lack of studies may be the difficulty of collecting climbers in dense forests, combined with the practical difficulties of collecting samples from the canopy (Putz 1984, Gentry 1991). Another may be that they are not timber species and thus considered to be of little importance for forestry (Arnold and Pérez 2001).

However, studies that specifically address the floristics and ecology of climbers have increased in the tropics (DeWalt et al. 2015). In Brazil, in particular, they are focused on semi-deciduous seasonal forest (e.g. Morellato and Leitão Filho 1998, Hora and Soares 2002, Udulutsch et al. 2004, Rezende and Ranga 2005, Santos et al. 2009, Udulutsch et al. 2010) or tropical rainforest (e.g. Lima et al. 1997, Oliveira et al. 2008). In areas occupied by savannah formations, there are only the studies of Batalha et al. (1997), Batalha and Mantovani (2001), Weiser (2007), Rossato et al. (2008) and Brito et al. (2017). Studies by Weiser (2007) and Brito et al. (2017) focus exclusively on lianas.

Here, we emphasise climbers in the broad sense, including both herbaceous and woody taxa, not only because of the role played by this life form in various biomes and vegetation types, but also because of the importance of the Estação Ecológica de Assis (EEA) for the conservation of cerradão fragments in the state of São Paulo. The EEA is the largest and richest area of continuous cerrado *sensu lato* in the state (Ratter et al. 2003).

Furthermore, considering the small number of studies including lianas in areas of cerradão, the two main objectives of our research were to make a floristic survey and to create identification keys for the 32 species of climbers found in the EEA.

## Methods

### Study area

**Figure 1.**
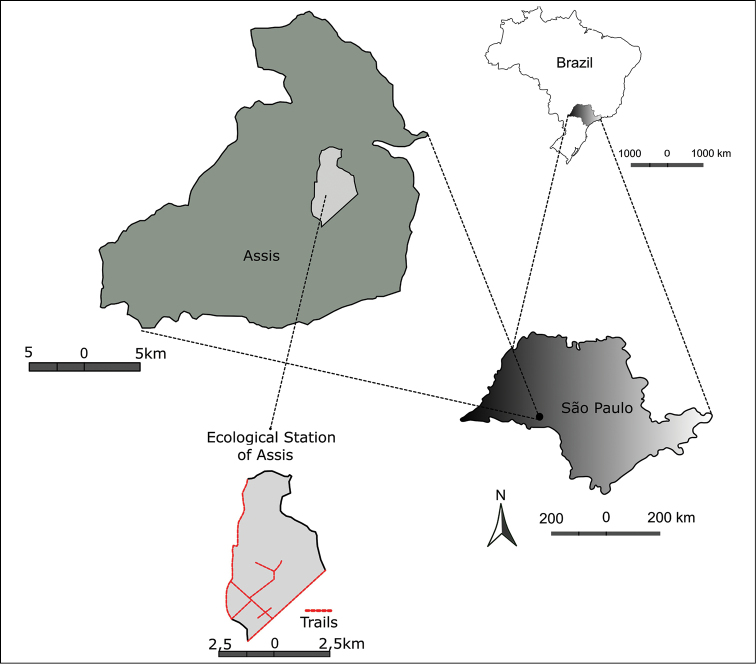
Study area, Estação Ecológica de Assis, state of São Paulo, Brazil (trails and edges sampled in red).

The Estação Ecológica de Assis (EEA) is located in the western region of the state of São Paulo (Figure 1), 12 km away from the Assis city centre and occupies an area of 1,760.64 ha. It is located between the coordinates 22°33'20" to 22°37'41"S and 50°24'48" to 50°21'27"W, has an altitude of 500–588 m, with a gently undulating relief (Rossato et al. 2008).

The EEA is located in the transition zone between Cfa and Cwa climates (Köppen 1948), climatic types that differ mainly in the length of the dry season. In the study area, rainfall is concentrated in the summer, with an average annual rainfall of approximately 1,400 mm and average temperatures of approximately 22 °C; during the winter, there is the possibility of severe frosts (Brando and Durigan 2004). The soils of the EEA are generally sandy, acidic and of low fertility (Rossato et al. 2008).

The vegetation of the EEA is characterised as cerrado *sensu lato* and there is a predominance of forested savannah (cerradão) physiognomy (Rossato et al. 2008). According to Batalha (2011), the EEA is in the seasonal forest biome.

### Sampling

We collected all climbing plants in fertile or sterile condition on trails and edges of forest (Figure 1), with collections made every two weeks over ten months from January to October 2013.

In addition, we recorded the climbing mechanisms by following the same criteria as Udulutsch et al. (2004, 2010): 1) Tendrilling: presence of tendrils and prehensile branches; 2) Apical twining: with twining stem; and 3) Scandent: with no specialised structure for climbing.

We prepared the herbarium samples according to standard techniques (Mori et al. 1989) and identified them by using specialised literature, such as revision studies and floras. Voucher specimens were deposited at HASSI (Universidade Estadual Paulista, UNESP) and SPSF (Instituto Florestal) herbaria.

Finally, we developed identification keys based exclusively on vegetative characters at both the family and species levels.

## Results

**Figure 2.**
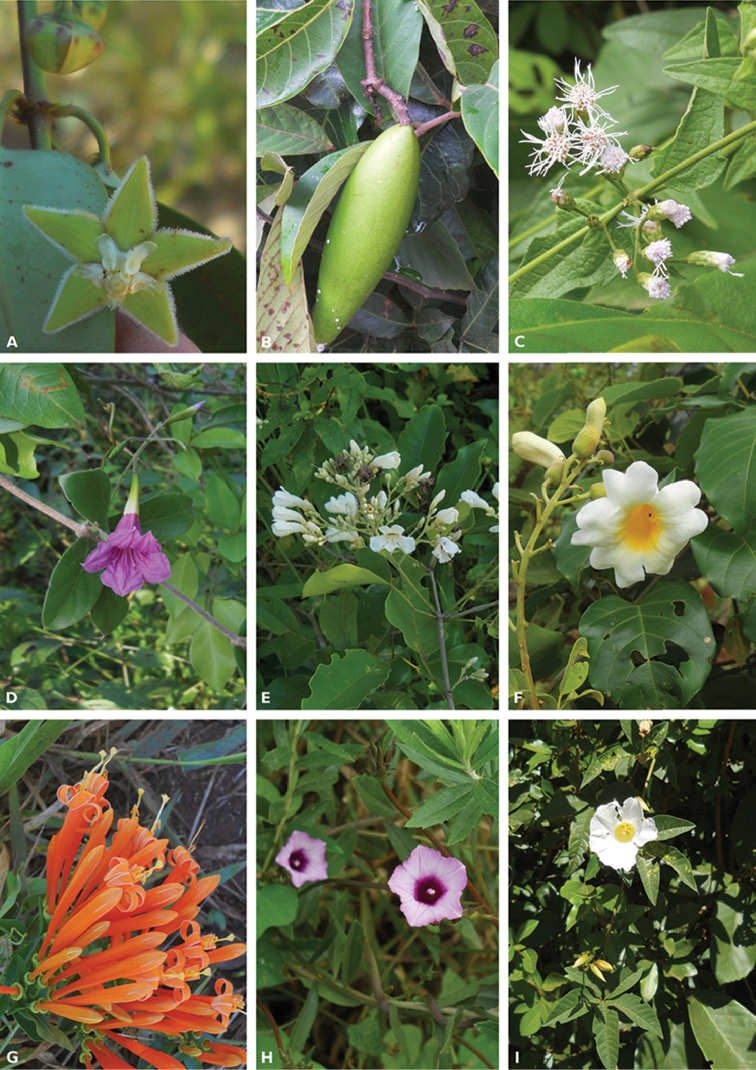
Apocynaceae (**A** *Blepharodon pictum* **B** *Odontadenia lutea*). Asteraceae (**C** *Chromolaena maximiliani*). Bignoniaceae (**D** *Fridericia craterophora* **E** *F. florida* **F** *Distictella mansoana* **G** *Pyrostegia venusta*). Convolvulaceae (**H** *Ipomoea aristolochiaefolia* **I** *Merremia macrocalyx*).

**Figure 3.**
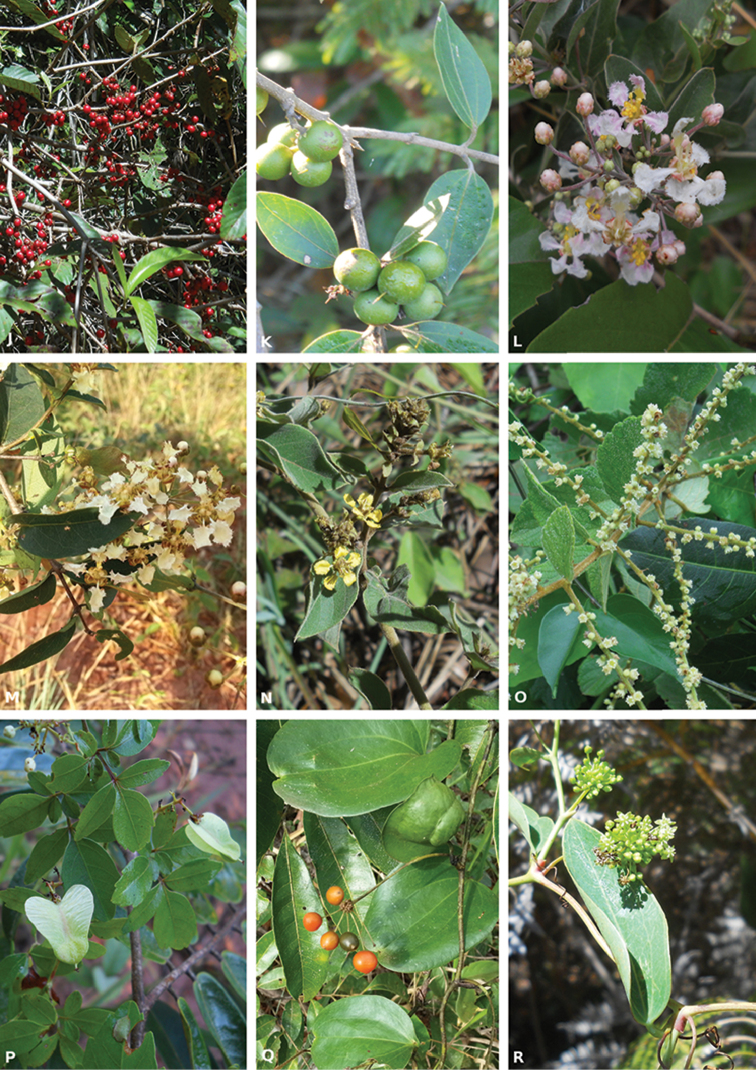
Dilleniaceae (J. *Doliocarpus dentatus*). Loganiaceae (**K** *Strychnos bicolor*). Malpighiaceae (**L** *Banisteriopsis muricata* **M** *B. stellaris* **N** *Mascagnia cordifolia*). Rhamnaceae (**O** *Gouania latifolia*). Sapindaceae (**P** *Serjania confertiflora*). Smilacaceae (**Q** *Smilax fluminensis*; R. *Smilax elastica*).

In this study, we found 32 species of lianas, which belong to 13 families and 24 genera (Table 1, Figures 2 and 3). Within the sampled families, the only two representatives of Monocotyledonae were Commelinaceae and Smilacaceae. The other 11 families were representatives of Eudicotyledonae and corresponded to 87.5% of the species. Amongst monocots and eudicots, the most speciose families were Bignoniaceae (eight species), followed by Malpighiaceae (five) and Apocynaceae and Smilacaceae (three species each) (Table 1). These four families represented 59.4% of the species sampled in the forest. The most genus-rich family overall was Bignoniaceae (with five genera present in EEA, Table 1).

The genera with the highest number of species were *Fridericia* (four species), followed by *Banisteriopsis* and *Smilax* (three species each). The remaining genera (83.3%) were represented by a single species. Overall, considering the morphology of the species, the most common combination of traits was woody habit and tendrilling climbing mechanism. We observed that about two-thirds of the species were woody (68.7%, 22 species) and one-third was herbaceous (31.3%, 10 species). Tendrilling climbers were predominant, representing 47% (15) of the species, followed by apically twining species with 44% (14) and then scandent forms with just 9.4% of all species (three) (Table 1).

**Table 1.** Climbers from the Estação Ecológica de Assis (SP, Brazil). Habit (H = herbaceous; L = woody), climbing mechanisms and voucher information (collector: Nicácio Ribeiro Neto, NRN; Raquel Aparecida Ronqui, RAR).

Family	Species	Habit	Climbing mechanisms	Collector number
Apocynaceae	*Blepharodon pictum* (Vahl) W.D. Stevens	H	apical twining	NRN 78
	*Odontadenia lutea* (Vell.) Markgr.	H	apical twining	NRN 16
	*Temnadenia violacea* (Vell.) Miers	H	apical twining	NRN 52
Asteraceae	*Chromolaena maximiliani* (Schrad. ex DC.) R.M. King & H. Rob.	L	scandent	NRN 20
	*Mikania hirsutissima* DC.	L	apical twining	NRN 51
Bignoniaceae	*Adenocalymma peregrinum* (Miers) L.G. Lohmann	L	tendrilling	NRN 32
	*Amphilophium mansoanum* (DC.) L.G. Lohmann	L	tendrilling	NRN 7, 36, 47
	*Cuspidaria convoluta* (Vell.) A.H. Gentry	L	tendrilling	NRN 3, 49
	*Fridericia craterophora* (DC.) L.G. Lohmann	L	tendrilling	NRN 21
	*Fridericia florida* (DC.) L.G. Lohmann	L	tendrilling	NRN 2, 53
	*Fridericia pulchella* (Cham.) L.G. Lohmann	L	tendrilling	NRN 6, 46
	*Fridericia samydoides* (Cham.) L.G. Lohmann	L	tendrilling	NRN 4, 15
	*Pyrostegia venusta* (Ker Gawl.) Miers	L	tendrilling	NRN 40, 77
Commelinaceae	*Dichorisandra hexandra* (Aubl.) C.B. Clarke	H	scandent	NRN 22
Convolvulaceae	*Ipomoea aristolochiifolia* G. Don	H	apical twining	NRN 10, 19
	*Merremia macrocalyx* (Ruiz & Pav.) O’Donell	H	apical twining	NRN 11, 12
Dilleniaceae	*Davilla elliptica* A. St.-Hil.	L	apical twining	NRN 45
	*Doliocarpus dentatus* (Aubl.) Standl.	L	scandent	NRN 5, 13, 48
Loganiaceae	*Strychnos bicolor* Progel	L	tendrilling	NRN 29
Malpighiaceae	*Banisteriopsis adenopoda* (A. Juss.) B. Gates	L	apical twining	NRN 41, 74
	*Banisteriopsis muricata* (Cav.) Cuatrec.	L	apical twining	NRN 1, 8
	*Banisteriopsis stellaris* (Griseb.) B. Gates	L	apical twining	NRN 25, 27, 28, 39
	*Heteropterys byrsonimifolia* A. Juss.	L	apical twining	NRN 44
	*Mascagnia cordifolia* (A. Juss.) Griseb.	L	apical twining	NRN 43, 75
Polygalaceae	*Securidaca divaricata* Nees & Mart.	L	apical twining	RAR 39
Rhamnaceae	*Gouania latifolia* Reissek	L	tendrilling	NRN 9, 17, 38, 50
Rubiaceae	*Manettia cordifolia* Mart.	H	apical twining	NRN 34
Sapindaceae	*Serjania confertiflora* Radlk.	L	tendrilling	NRN 24, 31, 33
	*Serjania lethalis* A. St.-Hil.	L	tendrilling	NRN 42
Smilacaceae	*Smilax campestris* Griseb.	H	tendrilling	NRN 30
	*Smilax elastica* Griseb.	H	tendrilling	NRN 76
	*Smilax fluminensis* Steud.	H	tendrilling	NRN 37

### Key to families of lianas from the Estação Ecológica de Assis^1^

**Table:** 

1	Leaves compound	**2**
–	Leaves simple	**4**
2	Leaves opposite	**Bignoniaceae (Key 3)**
–	Leaves alternate	**3**
3	Laeves biternate	**Sapindaceae (Key 7)**
–	Leaves palmately compound	**Convolvulaceae (*Merremia macrocalyx*)**
4	Leaves opposite	**5**
–	Leaves alternate	**9**
5	Latex present	**Apocynaceae (Key 1)**
–	Latex absent	**6**
6	Stipules present	**7**
–	Stipules absent	**8**
7	Stipules intrapetiolar; leaf nectaries present (blade or petiole)	**Malpighiaceae (Key 6)**
–	Stipules interpetiolar; leaf nectaries absent	**Rubiaceae (*Manettia cordifolia*)**
8	Tendrils present; leaf margin entire	**Loganiaceae (*Strychnos bicolor*)**
–	Tendrils absent; leaf margin serrate	**Asteraceae (Key 2)**
9	Petiolar tendrils present	**Smilacaceae (Key 8)**
–	Petiolar tendrils absent	**10**
10	Leaf venation parallel, basal sheath present	**Commelinaceae (*Dichorisandra hexandra*)**
	Leaf venation pinnate, basal sheath absent	11
11	Leaf margin pinnatifid or lobed	**Convolvulaceae (Key 4)**
–	Leaf margin entire	**12**
12	Leaves with conspicuous glands at the apex of secondary veins, near the margin of the blade	**Rhamnaceae (*Gouania latifolia*)**
–	Leaves without glands	**13**
13	Leaf venation craspedodromous (secondary veins ending in marginal teeth)	**Dilleniaceae (Key 5)**
–	Leaf venation brochidodromous (secondary veins looping)	**Polygalaceae (*Securidaca rivinaefolia*)**

### Key 1: Apocynaceae

**Table:** 

1	Leaves tomentose on both surfaces; latex watery	***Temnadenia violacea***
–	Leaves glabrous on both surfaces; latex white	**2**
2	Herbaceous climber; leaves with inconspicuous, non-prominent veins on abaxial surface	***Blepharodon pictum***
–	Woody climber; leaves with conspicuous, prominent veins on abaxial surface	***Odontadenia lutea***

### Key 2: Asteraceae

**Table:** 

1	Stems hirsute; leaves hirsute, cordate at base, caudate at apex	***Mikania hirsutissima***
–	Stems pubescent; leaves pubescent, cuneate at base, acute or acuminate at apex	***Chromolaena maximiliani***

### Key 3: Bignoniaceae

**Table:** 

1	Leaves biternate	***Adenocalymma peregrinum***
–	Leaves ternate	**2**
2	Tendrils trifid	**3**
–	Tendrils single	**4**
3	Stem costate; leaves abaxially pellucid-lepidote (glossy scales)	***Pyrostegia venusta***
–	Stem without costae; leaves abaxially tomentose, scales absent	***Amphilophium mansoanum***
4	Interpetiolar glands present	***Fridericia florida***
–	Interpetiolar glands absent	**5**
5	Leaf domatia absent	***Fridericia samydoides***
–	Leaf domatia present	**6**
6	Leaves subsessile, petiole about 1 mm long	***Fridericia craterophora***
–	Leaves petiolate, petioles more than 1 cm long	**7**
7	Domatia on secondary veins axils; prophyll of the axillary bud deciduous	***Fridericia pulchella***
–	Domatia on secondary and terciary veins axils; prophyll of the axillary bud persistent	***Cuspidaria convoluta***

### Key 4: Convolvulaceae

**Table:** 

1	Leaf entire or trilobed	***Ipomoea aristolochiifolia***
–	Leaf pinatissect and/or digitated	***Merremia macrocalyx***

### Key 5: Dilleniaceae

**Table:** 

1	Leaf blades with same colour on both surfaces when dried, adaxial face smooth	***Doliocarpus dentatus***
–	Leaf blades with different colours on adaxial and abaxial surfaces when dried, adaxial surface asperous	***Davilla elliptica***

### Key 6: Malpighiaceae

**Table:** 

1	Leaves abaxially covered with red-brown trichomes	***Heteropterys byrsonimifolia***
–	Leaves abaxially glabrous or with whitish indumentum	**2**
2	Nectaries (glands) on leaf margin	***Mascagnia cordifolia***
–	Nectaries (glands) in the basal portion leaf blade, abaxially	**3**
3	Leaves with white tomentum on abaxial surface	***Banisteriopsis muricata***
–	Leaves glabrous abaxially (indumentum only on petiole)	**4**
4	Nectaries between the petiole and the abaxial surface of leaf blade	***Banisteriopsis adenopoda***
–	Nectaries suprabasal between the basal and medial region of the abaxial surface of the leaf blade	***Banisteriopsis stellaris***

### Key 7: Sapindaceae

**Table:** 

1	Stems sharply 5-6-angled; leaf rachis terete	***Serjania confertiflora***
–	Stems terete or trigonous; leaf rachis winged or margined	***Serjania lethalis***

### Key 8: Smilacaceae

**Table:** 

1	Leaves cordate at base and emarginate or obtuse at apex	***Smilax fluminensis***
–	Leaves acute at base and apex	**2**
2	Cataphylls persistent	***Smilax elastica***
–	Cataphylls caducous	***Smilax campestris***

## Discussion

The number of species included in our survey reveals an important role of climbing plants in the plant diversity of forested savannahs (cerradão) in São Paulo. For example, Batalha and Mantovani (2001) and Rossato et al. (2008), working in the same vegetation type, presented similar number of species. In these studies, the contribution of climber species ranged from 15 to 21%. Considering only trees, shrubs and climbing plants, Weiser (2007) found 52 species of climbers out of 192 species (27% of the total). However, Weiser’s sampling effort was much greater than in most studies, as she covered a three-year interval, while the remaining studies were performed over one to one and a half years. Batalha et al. (1997) reported 49 species (40%) of climbers out of 121 species, however their sampling included several phytophysiognomies other than cerradão (campo-sujo, campo-cerrado and cerrado s.s.) and did not include herbs.

The high species richness of climbers in EEA is likely related to the following factors: 1) richness of this life form in tropical forests; 2) heterogeneity of habitats in which climbing plants thrive (Hora and Soares 2002); and 3) fragmentation of the environment (Morellato and Leitão Filho 1998). As fragmentation increases, the variation in the light also increases due to the increment in border area and the number of clearings. Climbers’ growth is favoured by a higher light incidence (Gentry 1991). Thus the density of climbers tends to increase over time, making them extremely competitive in situations of primary succession.

From a floristic point of view, in EEA most species (59.4%) were concentrated in four families (Bignoniaceae, Malpighiaceae, Apocynaceae and Smilaceae), corroborating the results of other floristic surveys conducted in tropical forests (e.g. Gentry 1988, Lima et al. 1997, Morellato and Leitão Filho 1998). Although the number of botanical families in which climbers occur is large (at least 97 families of angiosperms in the New World), the vast majority of species is concentrated in a few families, with 27 families accounting for 85% of the new world species (Gentry 1991).

In our study, Bignoniaceae includes the highest number of species of climbers (ca. 25% of all species), corroborating the results of previous surveys in seasonal forests in south-eastern Brazil (Hora and Soares 2002, Udulutsch et al. 2004, 2010, Rezende and Ranga 2005, Tibiriçá et al. 2006, Rezende et al. 2007), where this family has the highest species richness. Similarly, Apocynaceae, Malpighiaceae and Smilacaceae are families with higher species representation in EEA and they are amongst the most important families of climbers in other areas of Brazilian seasonal forests (Udulutsch et al. 2010).

The family Bignoniaceae has not only the largest number of species, but also the largest number of genera (five) and the genus with the highest number of species (*Fridericia*, with four species). This can be explained by the fact that 1) the cerradão physiognomy is a seasonal forest biome (Batalha 2011), where the Bignoniaceae is the most species-rich family; 2) the family Bignoniaceae is amongst the 10 families with the largest number of genera of climbers (21) in the Americas (Lohmann and Taylor 2014); and 3) Brazil is the centre of diversity for the family (Gentry 1980, 1991, Udulutsch et al. 2010).

On the other hand, the family Fabaceae was not sampled in our survey, although it is very often represented by a high number of species in floristic surveys of climbing plants in general (e.g. Barros et al. 2009, Brito et al. 2017). However, in other censuses of climbing species in areas of cerradão in São Paulo state, Rossato et al. (2008) found only one species of Fabaceae, while Batalha and Mantovani (2001) did not find any species of Fabaceae at all. Thus, our results are in agreement with those previous ones, indicating a low species diversity of climbing species of Fabaceae in cerradão in São Paulo. It is noteworthy, however, that, in the literature, the number of species of Fabaceae sampled seems to be related to 1) Sampling approach, whether the authors sampled one or several phytophysiognomies (Batalha et al. 1997, found 3 species); 2) Sampling effort (Weiser 2007, 4 species), whether the study was carried out in one or more years; and 3) Where the study was carried out, whether in the same state or not (Brito et al. 2017, 5 species).

Compared with surveys of lianas in seasonal semi-deciduous forests of the state of São Paulo, our results show that there is a small number of shared species with our species list, ranging from four (out of 45, Hora and Soares 2002) to 15 species (out of 148 species, Udulutsch et al. 2004). This may be due to differences in soil and altitude, factors that have been reported as important for the diversity of both climbers (Gentry 1988) and trees (Pagano et al. 1987). The studies by Hora and Soares (2002) and Udulutsch et al. (2004) were conducted in areas with clay soil and ca. 700 m altitude, while our study was undertaken in an area of sandy soil and ca. 544 m altitude. However, when comparing our results to others studies carried out in the same phytophysiognomy (cerradão), altitude range and soil type, the number of shared species ranged from 14 (out of 22, Batalha and Mantovani 2001) to 17 (out of 40, Rossato et al. 2008) species. Thus, these results reinforce that soil and altitude are important factors for the distribution of climbing species.

Similar to results from other studies in seasonal forests (Morellato and Leitão Filho 1998, Udulutsch et al. 2004), we found that, in the EEA, lianas represent about two thirds of the climbing species. This is in contrast, however, to results of some studies outside Brazil that have found herbaceous and woody climber species to be in approximately equal proportions (Croat 1978, Janzen and Liesner 1980, Gentry and Dodson 1987, Gentry 1991).

Given the recognised taxonomic and ecological significance of the climbing mechanisms of climbers, studies have generally quantified and classified these characteristics (e.g. Udulutsch et al. 2004, 2010, Tibiriçá et al. 2006, Brito et al. 2017). In the present study, the more passive scandent mechanism was present in only three species, demonstrating that the tendrillate (15 species) and apical twining (14) forms predominate, supporting the idea that specific climbing mechanisms may have contributed to the evolutionary success of this life form (Gentry 1991).

## Conclusion

The floristic composition of the studied area is similar to that of other fragments of forested savannahs and seasonal semi-deciduous forests in southern Brazil. This reinforces Batalha’s (2011) proposal that forest savannahs belong to the seasonal forest biome. However, this similarity is restricted to the genus level, because the composition at the species level is quite distinct. Due to few existing studies on cerradão physiognomy, little can be concluded about these floristic differences without further studies that examine population sizes and local distribution within habitats.

## Footnotes

This key should be used in the EEA, rather than in other areas of cerradão (forest savannah), as the species may differ.
